# Examining the effect of peer-support on self-stigma among persons living with HIV/AIDS

**DOI:** 10.11604/pamj.2019.34.200.17652

**Published:** 2019-12-16

**Authors:** Onyinye Hope Chime, Susan Uzoamaka Arinze-onyia, Edmund Ndudi Ossai

**Affiliations:** 1 Department of Community Medicine, Enugu State University Teaching Hospital, Enugu, Nigeria; 2 Department of Community Medicine, Enugu State University College of Medicine, Enugu, Nigeria; 3 Department of Community Medicine, Federal Teaching Hospital Abakaliki, Ebonyi, Nigeria

**Keywords:** Self-stigma, peer support, PLWHA, Enugu State, Nigeria

## Abstract

**Introduction:**

Contrary to assertions that stigma may no longer be experienced due to substantial advancement in antiretroviral access and treatment, self-stigma still exists among people living with HIV/AIDS (PLWHA) in Nigeria. The use of peer health workers to improve care in underserviced settings has been implemented by a number of programs, but its effect has not been extensively assessed. This study compared the level of self-stigma among PLWHA in peer support and non-support groups in Enugu State Nigeria.

**Methods:**

A cross-sectional study using quantitative and qualitative instruments was conducted among PLWHA attending ARV clinics. Quantitative data was assessed using pre-tested structured interviewer-administered questionnaires. Chi-square test of statistical significance was used in the analysis. Four focus group discussions and eight key informant interviews were conducted among PLWHA and healthcare workers respectively. Manual content analysis was used to analyse the qualitative data.

**Results:**

Comparable proportions of respondents in peer support (31.4%) and non-peer support groups (30.2%) had self-stigma (p=0.709). Disclosure was higher among respondents in non-peer support groups (96%) against 94.5% in peer support groups (p=0.331). Health workers interviewed were of the opinion that self-stigma cuts across members of both groups. Participants in the FGD reported that the media and economic empowerment have helped reduce self-stigma among PLWHA.

**Conclusion:**

Though peer support groups may be a starting place for the development of social support interventions for PLWHA, it might not be sufficient to combat self-stigma. Interventions aimed at economic empowerment of PLWHA and public enlightenment are essential for effective mitigation against self-stigma.

## Introduction

The World Health Organisation (WHO) cites fear of stigma as the main reason why people are reluctant to get tested, disclose their HIV status, take antiretroviral (ARV) drugs and access other antiretroviral therapy (ART) services [1,2]. HIV-related stigma and discrimination occurs when injustice, social disapproval and abuse are directed at people living with HIV/AIDS (PLWHA) [2]. The main reason why HIV positive women in sub-Saharan Africa fail to seek assistance and disclose their status is due to stigma [3]. Upon disclosure, PLWHA may lose employment, financial resources, and even family and friends as major sources of support and in some cases, suffer abandonment [4,5]. This may be due to the fact that HIV is primarily transmitted through sexual intercourse and people sometimes erroneously link HIV infection with promiscuity and infidelity [6]. The term self-stigma refers to a state when a patient isolates himself/herself due to personal convictions following diagnosis of ailment [7]. In Nigeria, as in other sub-Saharan African countries like Uganda, stigma remains a concern among PLWHA [8,9]. Prevalence of self-stigma ranged from 9.6% in Malawi to 45.0% in Burkina Faso and 33% to 66% among men and 23% to 61% among women in South Africa [10]. This has accounted for the emotional reactions and distress experienced by PLWHA resulting to self-condemnation, social drift and unwillingness to seek help or access available resources [3,4]. In light of the beliefs and the stigma that still surround the diagnosis and treatment of HIV, there is a growing interest in strategies that employ HIV positive peers who address these concerns and can serve as role models to others. In a study in Uganda, the collective activities of peer support groups provided practical skills to cope with external stigma and confidence to overcome self-stigma [11]. In the Nigerian context, while literatures have recognized the frequent use of support groups by PLWHA, few studies attempted to determine any difference in self-stigma among members of peer support groups and their counterparts not in any support group. There remains a gap in the knowledge; whether participation in peer support group activities reduces self-stigma among PLWHA in Nigeria.

## Methods

The study was conducted in Enugu State in the Southeast geopolitical zone of Nigeria. It has a population of 3,267,837 with an HIV prevalence of 4.9% [12,13]. ART services in Enugu are currently provided by PERPFAR agencies in both public and private health institutions in partnership with the Federal and State Ministries of Health. PLWHA support group members meet at least once a month and carry out activities related to positive prevention, stigma reduction, and group psychosocial therapy. Not all PLWHA enrolled in care participate in HIV support group activities. The study was an analytical cross-sectional facility-based study and compared findings among PLWHA belonging to peer-support groups and those not belonging to any support groups in Enugu State using both qualitative and quantitative study instruments. Study participants consisted of adult PLWHA enrolled in care and Healthcare workers (HCWs) in health facilities that provide comprehensive ART services including peer support services. A minimum of two attendances to monthly support group meetings in every quarter qualified a PLWHA for inclusion into peer support groups. A minimum sample size of 420 was estimated for each study group. A two-stage sampling method was used in the study. First, the fourteen health facilities in the state with functional facility-based peer support groups were stratified according to the three senatorial zones in the state. Simple random sampling technique was used to select eight health facilities proportionately. Participants were then stratified into those belonging to peer support groups and those not belonging to peer support groups.

The sampling frame for the study was determined using last attendance register for the three months preceding the study. The sample size for each group was proportionally allocated to the facilities based on the average number of patients currently receiving ART in each facility. Systematic sampling technique was used to select the study participants as they presented to the clinics. A total of 8 HCWs were purposively selected from the selected health facilities for key informant interview (KII). Simple random sampling technique was used to select four facilities from the selected health facilities for the focus group discussion (FGD). A total of four sessions were held; two for males and two for females. Eighteen males and twenty females were purposively recruited for the FGD. Quantitative and qualitative study instruments developed by the researchers in both English and 'Igbo' (the local language) were used for data collection. Self-stigma was assessed using an AIDS-Related Stigma Scale adapted from a similar study [3] on a 5-point Likert scale. The total scores ranged from 6 to 30. A score of 6 - 18 indicates low stigma, while 19 - 30 indicates high self-stigma. Data entry and analysis were done using Statistical Package for Social Sciences version 22. Chi-square test, student t-test, and bivariate analysis were used in the analysis. Level of statistical significance was determined by a p-value of < 0.05. Qualitative data were collected using pre-tested KII and FGD guide. Manual content analysis was used to analyse the qualitative data. The recorded interview was compared with written notes from the note taker for completeness, accuracy and quality assurance. The notes were then transcribed and coded. Identified themes that emerged from the sessions were grouped together under wider themes. Three themes emerged from the qualitative data.

**Ethical Considerations:** in compliance with the Helsinki Declaration, ethical approval for the study was obtained from the Health Research and Ethics Committee of University of Nigeria Teaching Hospital (UNTH) Enugu. Approval was also obtained from Enugu State Ministry of Health and the management of the selected health facilities. Participation in the study was voluntary. Written informed consents were obtained from the participants.

**Limitation:** having conducted the study on PLWHA in facility-based peer support groups, data on PLWHA who belong to community-based support groups were not captured in the study. This may limit generalisation of the study as this is not a clear representation of the population of PLWHA.

## Results

**Quantitative findings:** a total of 840 PLWHA participated in this study. Mean age of respondents in support group was 38.5±9.6 years while non-peer support group was 38.5±10.1 years. The highest proportion of the respondents in the two study groups were in the age group 30-39 years. A higher proportion of the respondents in the peer support group were females (80.2%) when compared with those in the non-peer support group, 72.4% (p=0.007) (Table 1). As reported in (Table 2), respondents reporting to hide their HIV status were more likely to belong to non-peer support group (87.4%) when compared with respondents from peer support group (80.5%). This finding was statistically significant (Χ^2^=7.423, p=0.006). Upon dichotomisation into low and high self-stigma, a comparable proportion of respondents in the peer support and non-peer support groups were classified as having high self-stigma (Χ^2^=0.140, p=0.709) (Table 2). Table 3 shows that majority of the respondents in the two study groups disclosed their HIV status to their partners, (support group=41.6% and non-support group, 45.4%) while the least proportion disclosed to religious leaders, (peer support group =0.5% and non-peer support group 0.7%) and the difference in proportions was not statistically significant (Χ^2^=4.834, p=0.565) (Table 3). In (Table 4), a higher proportion of respondents in the peer support group with low socioeconomic status (35.2%) were self-stigmatized when compared with those with high socioeconomic status, (27.5%) but the difference in proportions was not statistically significant, (Χ^2^=2.869, p=0.090). A significantly higher proportion of respondents in the non-peer support group who were in the low socio-economic status were self-stigmatized when compared with those who were in the high socio-economic status, (Χ^2^=4.421, p=0.035) (Table 4).

**Table 1:** Socio-demographic characteristics of respondents in peer and non-peer support groups

Variable	Support Group n=420; N (%)	Non-support Group; n=420; N (%)	χ^2^	p-value
**Age of respondents**				
Mean ±(SD)	38.5±9.6	38.5±10.1	0.063*	0.950
**Age in groups**				
<30 years	65 (15.5)	71 (16.9)	2.672	0.445
30 – 39 years	176 (41.9)	177 (42.1)		
40 – 49 years	123 (29.3)	105 (25.0)		
≥50 years	56 (13.3)	67 (16.0)		
**Gender**				
Female	337 (80.2)	304 (72.4)	7.171	0.007
Male	83 (19.8)	116 (27.6)		
**Marital status**				
Single	87 (20.7)	64 (15.2)	12.927	0.005
Married	206 (49.0)	239 (56.9)		
Separated/Divorced	3 (0.7)	12 (2.9)		
Widowed	124 (29.5)	105 (25.0)		
**Number of living children**				
None	105 (25)	93 (22.1)	2.744	0.254
1 – 4	250 (59.5)	245 (58.3)		
≥5	65 (15.5)	82 (19.5)		
**Education of respondent**				
No formal education	19 (4.5)	25 (6.0)	2.944	0.400
Primary education	167 (39.8)	145 (34.5)		
Secondary education	174 (41.4)	188 (44.8)		
Tertiary education	60 (14.3)	62 (14.8)		
**Employment status of the respondent**				
Self-employed	326 (77.6)	297 (70.7)	5.673	0.059
Salary Earners	54 (12.9)	65 (15.5)		
Unemployed/Student	40 (9.5)	58 (13.8)		
**Socio-economic status**				
Poorest	113 (26.9)	101 (24.0)	1.366	0.713
Very Poor	100 (23.8)	111 (26.4)		
The Poor	104 (24.8)	101 (24.0)		
Least Poor	103 (24.5)	107 (25.5)		
**Area of residence**				
Rural	278 (66.2)	275 (65.5)	0.048	0.827
Urban	142 (33.8)	145 (34.5)		
**Current living status**				
With partner and children	163 (38.8)	194 (46.2)	8.226	0.84
With children	117 (27.9)	98 (23.3)		
Alone	56 (13.3)	45 (10.7)		
With partner	46 (11.0)	35 (8.3)		
Significant others	38 (9.0)	48 (11.4)		
HIV Status of partner	n = 360	n = 382		
Unknown	124 (34.4)	117 (30.6)	1.234	0.540
Negative	111 (30.8)	124 (32.5)		
Positive	125 (34.7)	141 (36.9)		

* Student t-test

**Table 2:** Self-stigma of respondents in peer support and non-peer support groups

Variable	Support Group n=420 N (%)	Non-support Group n=420 N (%)	χ^2^	p-value
**Difficult to tell people about my HIV status**				
Yes	367 (87.4)	379 (90.2)	1.725	0.189
No	53 (12.6)	41 (9.8)		
**Being HIV positive makes me feel contaminated**				
Yes	92 (21.9)	76 (18.1)	1.905	0.168
No	328 (78.1)	344 (81.9)		
**Feel guilty that I am HIV positive**				
Yes	13933.1)	128 (30.5)	0.664	0.415
No	281 (66.9)	292 (69.5)		
**Ashamed that I am HIV positive**				
Yes	142 (33.8)	164 (39.0)	2.488	0.115
No	278 (66.2)	256 (61.0)		
**Feel worthless because I am HIV positive**				
Yes	91 (21.7)	93 (22.1)	0.028	0.867
No	329 (78.3)	327 (77.9)		
**Hide my HIV status from others**				
Yes	338 (80.5)	367 (87.4)	7.423	0.006
No	82 (19.5)	53 (12.6)		
**Stigma categorised**				
Low self-stigma	288 (68.6)	293 (69.8)	0.140	0.709
High self-stigma	132 (31.4)	127 (30.2)		

**Table 3:** Disclosure of respondents in peer support and non-peer support groups

Variable	Support Group n=420 N (%)	Non-support Group n=420 N (%)	χ^2^	p-value
**Disclosed HIV status**				
Yes	397 (94.5)	403 (96.0)	0.945	0.331
No	23 (5.5)	17 (4.0)		
**Disclosure of HIV status to others**	n=397	n=403		
Partner	165 (41.6)	183 (45.4)	4.834	0.565
Sibling	90 (22.7)	86 (21.3)		
Child	54 (13.6)	44 (10.9)		
Relative	46 (11.6)	41 (10.2)		
Parents	33 (8.3)	32 (7.9)		
Friend	7 (1.8)	14 (3.5)		
Religious leader	2 (0.5)	3 (0.7)		

**Table 4:** Factors affecting self-stigma in peer support and non-peer support groups

Variable	Support Group		χ^2^	p-value	Non-Support Group		χ^2^	p-value
	Stigma Yes	Stigma No			Stigma Yes	Stigma No		
**Age in groups**								
<40 years	75 (31.1)	166 (68.9)	0.025	0.875	79 (31.9)	169 (68.1)	0.750	0.386
≥40 years	57 (31.8)	122 (68.2)			48 (27.9)	124 (72.1)		
**Gender**								
Female	108 (32.0)	229 (68.0)	0.303	0.582	91 (29.9)	213 (70.1)	0.048	0.826
Male	24 (28.9)	59 (71.1)			36 (31.0)	80 (69.0)		
**Marital status**								
Single	33 (25.0)	54 (18.8)	6.502	0.090	25 (19.7)	39 (13.3)	4.022	0.259
Married	69 (52.3)	137 (47.6)			73 (57.5)	166 (56.7)		
Separated/Divorced	0 (0.0)	3 (1.0)			3 (2.4)	9 (3.1)		
Widowed	30 (22.7)	94 (32.6)			26 (20.5)	79 (27.0)		
**Educational level**								
No formal education	6 (4.5)	13 (4.5)	2.682	0.443	11 (8.7)	14 (4.8)	3.172	0.366
Primary Education	58 (43.9)	109 (37.8)			39 (30.7)	106 (36.2)		
Secondary Education	54 (40.9)	120 (41.7)			59 (46.5)	129 (44.0)		
Tertiary Education	14 (10.6)	46 (16.0)			18 (14.2)	44 (15.0)		
**Employment status**								
Self-employed	102 (31.3)	224 (68.7)	0.024	0.988	95 (32.0)	202 (68.0)	2.780	0.249
Salary Earners	17 (31.5)	37 (68.5)			14 (21.5)	51 (78.5)		
Unemployed/student	13 (32.5)	27 (67.5)			18 (31.0)	40 (69.0)		
**Number of living children**								
None	34 (32.4)	71 (67.6)	0.113	0.945	31 (33.3)	62 (66.7)	0.609	0.738
1 – 4	77 (30.8)	173 (69.2)			71 (29.0)	174 (71.0)		
≥5	21 (32.3)	44 (67.7)			25 (30.5)	57 (69.5)		
**Area of residence**								
Rural	95 (34.2)	183 (65.8)	2.873	0.090	88 (32.0)	187 (68.0)	1.172	0.279
Urban	37 (26.1)	105 (73.9)			39 (26.9)	106 (73.1)		
**Socio-economic status (SES)**								
Low (SES)	75 (35.2)	138 (64.8)	2.869	0.090	74 (34.9)	138 (65.1)	4.421	0.035
High (SES)	57 (27.5)	150 (72.5)			53 (25.5)	155 (74.5)		

**Qualitative findings:** three themes emerged and they include; the position of self-stigma, disclosure of HIV status and measures to diminish self-stigma.

**Status of self-stigma:** all the persons interviewed accepted the fact that stigma is a big issue as far as HIV/AIDS is concerned, affecting all PLWHA including those in peer support and non-peer support groups. An ART coordinator said, *“ Yes they all have stigma, both those in support and non-support groups, to a large extent they have a feeling of shame and many of them find it difficult to overcome this stigma”*. Another adherence counselor went further to give reasons why stigma has been associated with HIV/AIDS, *“ Stigma is a killer, it actually means people distancing themselves from the beginning. Other diseases run a chronic course, so understanding is a very big factor, but I think the name we gave the disease (HIV) in the native language started it all, obiri na aja ocha thus implying that anybody with the disease is on the path of death, also there was the impression from the onset that anybody with the disease was wayward.”* Another HCW was of the opinion that most times stigma is self-inflicted and gave the impression that one can overcome it. He bared his mind this way, *“ Nothing kills quicker like self-stigma. Sometimes they are not stigmatised in any form. If they come to understand and accept their condition, they can face anything. Afterall, they are not the only ones with the disease”.* An adherence counselor differentiated stigma based on duration on ARV. According to him, those who have been on treatment for shorter duration are the ones that are mostly self-stigmatised. The participants from non-peer support groups complained of long waiting time when they come to clinics. While the HCWs attributed the long waiting time to a large number of clients they have to attend to, the participants from non-peer support group linked the long waiting time to stigma as it exposes the patients to people who may know them.

**Disclosure of HIV status:** opinions on disclosure of HIV status to others were varied. An HCW believes that disclosure of HIV status helps to eliminate self-stigma. This according to him is based on the fact that HIV does not kill again as before hence people are not afraid anymore to disclose their status. He went further to compare disclosure of HIV status among the peer support and non-peer support group; *“ The PLWHA who do not belong to support group lack knowledge and boldness unlike those in the support group hence they do not disclose their status like their counterparts in the support groups ”*. This concept of boldness was corroborated by a female participant in non-peer support FGD. She was of the opinion that she lacked boldness and because of this she has a prize to pay, *“ I am not bold when I come to the hospital. It´s like everyone knows why I´m at the hospital and all eyes are on me. I feel so ashamed ”*. Another female participant from non-peer support group declared she had no shame but her confidence was based on the way she got infected with the virus, *“ I have no shame. I did not get it from sexual intercourse but from a nurse during childbirth. My husband knows about it and although he is negative, he has no issues with it ”*. However, a male participant (peer support group) had a different view, for him disclosure begets stigma; *“ I didn´t disclose my status to anyone so that I can relate freely with everyone. The point is that if I do not tell you that I have HIV, you will not know ”* It appears also that there is a prize to pay for disclosure as according to a female participant in non-peer support group FGD, *“ My mum is aware of my status. She asked me one day to have my own soap dish and I felt bad, but my mother feels it is for my good and the good of the family ”*. Another female participant in the non-peer support group had a remarkable experience after her disclosure, *“ After my husband´s death, it was then I realised I had HIV. I disclosed to my brother in law who later spread the news to the extended family, even to my own family. I felt disappointed and regret having disclosed to anyone at all. It was a horrible experience ”*. It could be in a bid to avoid these forms of interferences that another female participant in non-peer support group FGD refused to disclose her status to her parents but her experiences have not been good based on that. She said, *“ I see a man that may want to marry me and I feel weak because HIV test must be done along the line, moreover my parents do not know my status, every day my mother reminds me that I should have married by now ”*.

**Measures to diminish self-stigma:** there were varied views on how to reduce self-stigma among PLWHA. However, most respondents were conscious of the fact that it should begin with the patients themselves. This supports the initial views of an adherence counselor who perceived stigma as being self-inflicted. A male participant in peer support group FGD had this to say, *“There is no disease that cannot kill a human be it even typhoid fever and cholera. What I have learned is to rejoice and believe that by rejoicing I will live my life to the full”* Another male participant from the peer support group was philosophical in his approach. His views were captured this way, *“ Nobody knows tomorrow and everyone should fear God, who knows the disease that may emerge tomorrow, it could be worse than HIV and who knows those that will suffer from it, so it is better for everyone to accord respect to all human beings.”* Most of the participants were of the opinion that being economically empowered reduces stigma. To a large extent, most of the participants believed that the government is capable of providing them with vocational training and the needed take-off grants. Some participants frowned at designating separate clinic areas for them as such clinics evoke curiosity among other regular users of the facility who will be eager to know why they are treated separately. Another male participant belonging to a peer support group was full of courage and also hope, he remarked, *“ Some diseases are worse than HIV, some people have kidney problems and some even suffer from cancer, so since I discovered that there are drugs for this disease (HIV) and the drugs are working, I am happy and I hope that one day a final cure will be discovered ”*. A male participant from the non-peer support group, however, relied on the implementation of the existing law against people who discriminated against PLWHA as a way of reducing or eliminating stigma. Another participant from the non-peer support group who perceived the good works the media have done in this direction wished that the media campaigns be intensified as that will reduce stigma completely. A female participant belonging to peer support group was however of the opinion that belonging to a peer support group is a good way of overcoming stigma. She said those that do not belong to a peer support group have no foundation and good knowledge of the disease hence are self-stigmatized. A female participant from a non-peer support group wrapped it up this way. *“ It starts from us, once we accept the situation as they are, then other things will certainly go on well and with that other peoples´ feelings will not matter anymore”*. From the views of a female participant in the non-peer support group, there seem to be some good sides to stigma, *“ My in-laws look at me like I will die today. If I come they do not like giving me food or for me to touch their spoons. What I do is to always take my drugs as I have seen that it is capable of preventing death and as you can see, I look healthy”*.

## Discussion

Self-stigma among PLWHA was found to be persistent among all PLWHA in this study as a comparable proportion of the respondents in peer support (31.4%) and non-peer support (30.2%) groups have high self-stigma. Health workers interviewed were of the opinion that stigma affects PLWHA equally. Aransiola *et al.* reported a similar finding in Southwest Nigeria where there was no change in the level of stigma for those in support groups when compared to their counterparts, not in support groups [6]. Similarly, self-stigma did not significantly differ after an intervention period of 12 months in a randomised controlled trial assessing the effect of peer support on quality of life and internal stigma among PLWHA in Vietnam [14]. Thus it can be argued that due to the complexity of self-stigma, the influence of peer support might not yield positive results within a short duration of the intervention. However, mixed findings were made in the FGD; while some participants corroborated the fact that participation in support group gives boldness which inadvertently overcomes stigma, others resolved that belonging to support group begets stigma. Studies in Uganda and Vietnam among PLWHA were however emphatic that interactions between PLWHA and their peers in support groups was effective in reducing stigma while respondents reporting self-stigma in a study conducted in four African countries were less likely to belong to support groups [10,11,15].

Disclosure is encouraged in support groups. Previous studies have reported that support-group participation assists with disclosure resulting to potential prevention benefits [16,17]. Respondents not belonging to support groups were found to lack knowledge and boldness unlike those in the support group hence had great difficulties disclosing their status unlike their counterparts in the support groups. In some instance, PLWHA join support groups due to experiences of discrimination encountered after disclosure of their status [10]. It is rather surprising that a higher proportion of respondents in the non-peer support groups (96.0%) disclosed their HIV status more than those belonging to peer support groups (94.5%) although this difference was not statistically significant. There are possible explanations for this disparity in disclosure. First, it could be suggested that peer support group participation may be a coping mechanism for PLWHA due to increased level of self-confidence as well as social benefits. This might be lacking in respondents not in support groups as they may need support from others hence the need to disclose to them. Similar findings were made by Madiba *et al.* in South Africa, where disclosure was found not to be associated with HIV support group participation as participants believed that since they were coping well, they did not need to belong to support groups [18]. However, the study is limited in that majority of the participants had never attended any support group and poor attendance to meetings was also reported in majority of support group attendees [18]. Secondly, the negative ordeals recounted by PLWHA after disclosure during support group meetings can serve as a deterrent to others, as they may fear possible discrimination following disclosure. HIV-positive women in Africa, Asia, and the United States have reportedly experienced violence after disclosing their HIV status to others [10].

Socio-economic status was found to significantly influence self-stigma. As respondents in the non-peer support group might not have additional social support, it is not surprising that a higher proportion of respondents with low socio-economic status were self-stigmatised when compared with those with high socioeconomic status. Most of the participants in the FGD were of the opinion that being economically empowered has the propensity to reduce self-stigma as it may upturn the self-worth of the individual. In Uganda, being wealthy appeared to cushion effect of stigma among PLWHA, especially men. PLWHA in support groups through collective efforts, engage in income-generating ventures to reduce poverty and indirectly counter stigma [11]. This pooling of labour and resources is a distinctive advantage of a “group” approach [11,15,19,20]. Women, in a bid to cope with stigma and discrimination, join and participate in HIV/AIDS support groups which have emerged in response to the AIDS epidemic [15,20]. However, membership in support groups is not sufficient alone to eliminate stigma, as shown by the findings from the study by Mburu *et al.* [11], rather multiple approaches are required such as sensitization training for teachers, health service providers, law enforcement personnel and others, for an effective multi-sectoral mitigation of HIV stigma. Similar to findings from another Nigerian study, participants during the FGD reported that delays at the health facilities have been linked to stigma as the long waiting time exposes the patients to being identified at the ART clinics by people who may know them [6]. The reason for this delay varies among the facilities; in some facilities, the waiting hall was small hence patients had to hang around the hall, thereby compounding their fears of being seen by others while some other facilities were understaffed increasing time spent in the clinics. Other participants complained that designated and specific clinics being assigned to PLWHA in their facilities is also a form of stigma.

## Conclusion

Though peer support groups may be used as a starting place for the development of social support interventions for PLWHA, it might not be sufficient to combat self-stigma in the study area. The current findings have important implications for services and interventions for people living with HIV/AIDS in Nigeria. ART services should be integrated into routine daily clinics and not run separately on special days or at designated clinics to reduce stigma. Government and donor agencies should intensify public enlightenment programs through the media as a means of mitigating against self-stigma. Anti-discrimination laws should be fully enforced.

### What is known about this topic

- Stigma and discrimination remain the main reason why people are reluctant to get tested, disclose their HIV status and access other ART services;
- It has been demonstrated as an effective intervention to overcome barriers to adherence in resource poor settings.

### What this study adds

- Self-stigma is persistent among PLWHA irrespective of whether they belong to peer support groups or not;
- Participation in peer-support groups did not enhance disclosure among PLWHA.

## Competing interests

The authors declare no competing interests.
